# Clinical Profile, Pharmacological Treatment, and Predictors of Death Among Hospitalized COVID-19 Patients With Acute Kidney Injury: A Population-Based Registry Analysis

**DOI:** 10.3389/fmed.2021.657977

**Published:** 2021-06-15

**Authors:** Eduardo Gutiérrez-Abejón, Débora Martín-García, Eduardo Tamayo, F. Javier Álvarez, Francisco Herrera-Gómez

**Affiliations:** ^1^Pharmacological Big Data Laboratory, Faculty of Medicine, University of Valladolid, Valladolid, Spain; ^2^Technical Direction of Pharmaceutical Assistance, Gerencia Regional de Salud de Castilla y León, Valladolid, Spain; ^3^Department of Nephrology, Hospital Clínico Universitario de Valladolid, Valladolid, Spain; ^4^BioCritic. Group for Biomedical Research in Critical Care Medicine, Valladolid, Spain; ^5^Department of Anaesthesiology, Hospital Clínico Universitario de Valladolid, Valladolid, Spain; ^6^Department of Surgery, Faculty of Medicine, University of Valladolid, Valladolid, Spain; ^7^Comité de Ética de la Investigación con medicamentos, Hospital Clínico Universitario de Valladolid, Valladolid, Spain; ^8^Unit of Kidney Resuscitation and Acute Purification Therapies, Hospital Virgen de la Concha, Zamora, Spain; ^9^Transplantation Center, Lausanne University Hospital & University of Lausanne, Lausanne, Switzerland

**Keywords:** SARS-CoV-2, COVID-19, acute kidney injury, chronic kidney disease, treatment, mortality

## Abstract

**Introduction:** One of the worst clinical outcomes of the coronavirus disease 2019 (COVID-19) pandemic was acute kidney injury (AKI).

**Methods:** This manuscript presents results from a population-based registry study assessing treatment, comorbidities, and predictors of hospital death among COVID-19 patients with AKI from March 1st to May 31th, 2020. Death, oxygen delivery and ventilation, acute dialysis need, use of medications, and various clinical outcomes, in addition to the length of stay in the hospital and intensive care unit (ICU), were evaluated.

**Results:** In Castile and Leon, the largest region of Spain, 10.87% of the patients admitted for COVID-19 (*n* = 7,307) developed AKI. These patients were known by having hypertension (57.93%), cardiovascular disease (48.99%), diabetes (26.7%) and chronic kidney disease (14.36%), and they used antibiotics (90.43%), antimalarials (60.45%), steroids (48.61%), antivirals (33.38%), anti-systemic inflammatory response syndrome (SIRS) drugs (9.45%), and tocilizumab (8.31%). Mortality among patients with AKI doubled that observed in patients without AKI (46.1 vs. 21.79%). Predictors of hospital death in COVID-19 patients with AKI were ventilation needs (OR = 5.9), treatment with steroids (OR = 1.7) or anti-SIRS (OR = 2.4), severe acute respiratory syndrome (SARS) occurrence (OR = 2.8), and SIRS occurrence (OR = 2.5).

**Conclusions:** Acute kidney injury is a frequent and serious complication among COVID-19 patients, with a very high mortality, that requires more attention by treating physicians, when prescribing medications, by looking for manifestations particular to the disease, such as SARS or SIRS.

## Introduction

Acute kidney injury (AKI) continues to affect between 10 and 40% of in-hospital coronavirus disease 2019 (COVID-19) patients (1–4). Since the beginning of the COVID-19 pandemic, mostly elderly individuals with many comorbidities have developed AKI and died (5, 6). The adaptation of mechanisms to the kidneys that respond to hemodynamic changes, inflammation, and other stress-inducing situations, perform worse in cases of previous kidney affectation, diabetes, heart failure, etc. Furthermore, in COVID-19 patients, systemic inflammatory response syndrome (SIRS) and severe acute respiratory syndrome (SARS) may exhaust kidney function capacities, leading to the appearance of AKI (4).

Acute kidney injury is a direct result of COVID-19 infection (7) and is common in critically ill patients, being one of the poor clinical outcomes with a negative prognosis for survival (4, 8).

In addition, AKI incidence and death rates are changing throughout the regions of world, probably in relation to the characteristics of the individuals in those regions. In this sense, we report our pharmacological, clinical, and epidemiological findings related to the in-hospital COVID-19 patients with AKI.

The main aim of this study was to describe the pharmacological treatment and the clinical baselines of the in-hospital COVID-19 patients affected by AKI (March 1st to May 31th, 2020), in Castile and Leon, the largest region of Spain. Furthermore, we have analyzed the risk factors associated with deaths of COVID-19 patients with AKI. Finally, the influence of AKI on the survival of the in-hospital COVID-19 patients was analyzed.

## Methods and Materials

### Real-World Study Details

This article presents findings from an epidemiological analysis carried out following a population-based registry study design, with the collection of clinical and administrative data. The Reporting of studies Conducted using Observational Routinely-collected health Data (RECORD) recommendations (9) and the Strengthening the Reporting of Observational Studies in Epidemiology (STROBE) standards (10) were adhered to. Ethics committee approval reference and date: PI 20-1863, June 11th, 2020.

This study evaluated cross-sectionally clinical findings, treatment, and outcomes from a population of COVID-19 patients with AKI. These study participants were selected from the total COVID-19 patient population with a recorded stay in public Castile and Leon hospitals between March 1st and May 31th, 2020. COVID-19 was diagnosed by in-hospital treating physicians who decided on hospitalizing the patients on the basis of clinical or radiological findings defining SARS (Supplementary Table 1). A positive result on the COVID-19 real-time reverse transcription polymerase chain reaction (rRT-PCR) test for qualitative detection of the nucleic acid from severe acute respiratory syndrome coronavirus 2 (SARS-CoV-2) was not required to formally diagnose the disease. SIRS was diagnosed following the proper criteria (Supplementary Table 1) (11).

### Definitions and Data Sources

Acute kidney injury was diagnosed in the hospital by treating physicians using the Kidney Disease Improving Global Outcomes (KDIGO) criteria (12) and, following these recommendations, by calculating glomerular filtration rate (GFR) with the Modification of Diet in Renal Disease (MDRD) study equation, or the Chronic Kidney Disease EPIdemiology Collaboration (CKD-EPI) equation (Supplementary Table 1): briefly, an increase in serum creatinine (SCr) by ≥0.3 mg/dL within 48 h or ≥1.5 times within the prior 7 days, in addition to an urine volume <0.5 ml/kg/h for 6 h, was required.

These patients may have chronic kidney disease (CKD), categories 3–5, defined by an estimated GFR of 60 ml/min or lower (13): the MDRD or CKD-EPI equations were used for following up the kidney function of patients with visits to Nephrology units, and the Cockcroft-Gault formula was used for patients with visits to primary healthcare centers depending on public Castile and Leon hospitals. Dialysis patients were excluded.

Cardiovascular disease was defined by the occurrence of major adverse cardiovascular events (MACE), which included all non-fatal coronary events, including revascularization procedures, and all cerebrovascular events, including transient ischemic attacks (TIA). Peripheral artery disease and decompensated heart failure (HF) were considered indicative of cardiovascular disease. Diabetes, hypertension, and other well-known cardiovascular risk factors were also considered.

Access to registries containing EHR information from the Castile and Leon hospitals and associated primary healthcare centers (Jimena and Medora, https://www.saludcastillayleon.es/sanidad/cm), hospital pharmaceutical care information in our region (Concylia, http://www.saludcastillayleon.es/portalmedicamento/es/indicadoresinformes/concylia) and hospital discharges information in Castile and Leon (Pestadistico, https://pestadistico.inteligenciadegestion.mscbs.es/publicoSNS/N/rae-cmbd/rae-cmbd), was obtained.

### Variables

The main outcome during the study period was death (March 1st to May 31th, 2020). Other outcomes were stays in the hospital and intensive care unit (ICU) (length in days), the need for acute dialysis, SARS, SIRS, disseminated intravascular coagulation (DIC), cardiomyopathy, and bacterial and fungal superinfection.

The use of medications to treat COVID-19 according to the Spanish national recommendations (14, 15) (Supplementary Table 2) during the study period (i.e., antibiotics, antimalarials, steroids, antivirals, tocilizumab, and other anti-SIRS), was assessed through dispensaries in public hospitals in Castile and Leon. Anatomical Therapeutic Chemical (ATC) classification was used to evaluate medication consumption (Supplementary Table 3). Data on the use of oxygen delivery using low-flow systems (nasal cannula and simple face masks) and high-flow systems (high-flow nasal cannula, venturi masks, and rebreather masks), non-invasive pressure positive ventilation (NIPPV), and invasive ventilation (IV), was also assessed.

### Statistical Analysis

Statistical analysis was carried out considering age and gender distributions and an age cut-off at 65 years of age. A 15-day period analysis was performed for all measurements and consideration of all clinical outcomes (March 1–14 to May 15–31, 2020). Frequencies (in percentages) and their corresponding 95% confidence intervals (95% CI) and means or medians with, respectively, their standard deviations (SD) or interquartile ranges (IQR), are presented, as appropriate.

Comparisons were performed using the Student *t-*test or the Mann-Whitney *U-*test (for continuous variables), after confirmation of normal distribution of data in a given variable (Kolmogorov-Smirnov-test), and using Pearson's chi-square-test or Fisher's exact-test (categorical variables).

Multiple logistic regression, with a forward selection approach, was performed for in-hospital COVID-19 patients with or without AKI, who died, as opposed to those who did not die. The odds ratio (OR) and a 95% CI were presented. The following variables were included in the analysis: age (>65 years), gender, comorbidities (hypertension, diabetes, cardiovascular disease, CKD), obesity (BMI ≥ 30 kg/m^2^), need for ventilation, acute dialysis need (only in patients developing AKI), medications used (antibiotics, antimalarials, steroids, antivirals, tocilizumab, or anti-SIRS), occurrence of SARS, SIRS, DIC, cardiomyopathy, and bacterial and fungal superinfections.

The survival of COVID-19 patients with and without AKI was performed using the Kaplan-Meier approach and the log-rank-test for comparison between groups.

The level of significance was set at *p* < 0.05. All statistical analyses were performed by using the Statistical Package for the Social Sciences (SPSS) software version 24.0. (SPSS Inc., Chicago, IL).

## Results

### Clinical Findings

From a total population of 7,307 in-hospital COVID-19 patients for which data were available, the findings presented here describe 794 patients with AKI (10.87%), most of them males aged 65 years or more. Around half of the patients had hypertension or cardiovascular disease (Table 1). Many of these patients also had diabetes mellitus (26.7%) and CKD categories 3–5 (14.36%).

**Table 1:** Baseline characteristics, treatment, and clinical outcomes of in-hospital COVID-19 patients with acute kidney injury in Castile and Leon (Spain) (March 1st–May 3th 2020).

	**Total**	**AKI**	**No. of AKI**	***p***
***N***	7,307	794	6,513	
Age (median and IQR)	76 (63–86)	84 (75–89)	75 (62–85)	0.001
Age < 65 (95% CI)	27.23 (26.21–28.25)	8.19 (6.28–10.09)	29.56 (28.45–30.67)	0.001
Age ≥ 65 (95% CI)	72.77 (71.75–73.79)	91.81 (89.91–93.72)	70.44 (69.33–71.55)	0.001
**Chronic diseases (95% CI)**				
Hypertension	43.74 (42.6–44.88)	57.93 (54.5–61.37)	42.01 (40.81–43.21)	0.001
Cardiovascular disease	35.83 (34.73–36.93)	48.99 (45.52–52.47)	34.22 (33.07–35.38)	0.001
Diabetes	18.9 (18–19.8)	26.7 (23.62–29.78)	17.95 (17.02–18.88)	0.001
Chronic kidney disease	5.9 (5.36–6.44)	14.36 (11.92–16.8)	4.87 (4.34–5.39)	0.001
**Treatment**				
**Oxygen delivery and ventilation (95% CI)**				
IV	3.5 (3.08–3.93)	10.2 (8.1–12.31)	2.69 (2.29–3.08)	0.001
Oxygen delivery	2.52 (2.16–2.88)	2.52 (1.43–3.61)	2.52 (2.14–2.9)	0.999
NIPPV	1.63 (1.34–1.92)	1.76 (0.85–2.68)	1.61 (1.31–1.92)	0.751
**Drugs (95% CI)**				
Antibiotics	90.83 (90.17–91.49)	90.43 (88.38–92.47)	90.88 (90.18–91.58)	0.677
Antimalarial	69.74 (68.69–70.79)	60.45 (57.05–63.85)	70.87 (69.77–71.98)	0.001
Steroids	44.37 (43.23–45.51)	48.61 (45.14–52.09)	43.85 (42.65–45.06)	0.011
Antivirals	42.63 (41.52–43.93)	33.38 (30.1–36.66)	43.79 (42.58–44.99)	0.001
Tocilizumab	9.37 (8.71–10.04)	8.31 (6.39–10.23)	9.5 (8.79–10.22)	0.277
Other anti-SIRS^*^	7.34 (6.74–7.93)	9.45 (7.41–11.48)	7.05 (6.43–7.67)	0.007
**Clinical outcomes**				
Hospital LoS (median and IQR)	9 (5–15)	10 (5–18)	9 (5–14)	0.001
ICU LoS (median and IQR)	15 (7–30)	13 (7–22)	16 (7–33)	0.271
***N***	491	99	392	
Death (95% CI)	24.43 (23.44–25.41)	46.1 (42.63–49.56)	21.79 (20.79–22.79)	0.001
Acute dialysis (95% CI)	0.95 (0.73–1.17)	3.9 (2.56–5.25)	0.6 (0.41–0.79)	0.001
SARS (95% CI)	14.03 (13.23–14.82)	24.18 (21.2–27.16)	12.79 (11.98–13.6)	0.001
Bacterial superinfection (95% CI)	3.59 (3.16–4.01)	13.85 (11.45–16.26)	2.33 (1.97–2.7)	0.001
Fungal superinfection (95% CI)	2.23 (1.89–2.57)	5.92 (4.28–7.56)	1.78 (1.46–2.1)	0.001
SIRS (95% CI)	2.22 (1.88–2.56)	10.71 (8.55–12.86)	1.77 (1.45–2.09)	0.001
Carodiomyopathy (95% CI)	1.15 (0.91–1.39)	2.9 (1.73–4.06)	0.94 (0.7–1.17)	0.001
DIC (95% CI)	0.18 (0.08–0.27)	1.01 (0.31–1.7)	0.08 (0.01–0.14)	0.001

* *Anakinra, baricitinib, interferon, ruxolitinib, siltuximab*.

*CI, confidence interval; IQR, interquartile range; AKI, acute kidney injury; IV, invasive ventilation; NIPPV, Non-invasive positive pressure ventilation; SIRS, systemic inflammatory response syndrome; ICU, intensive care unit; LoS, length of stay; SARS, severe acute respiratory syndrome; DIC, disseminated intravascular coagulation*.

Into the group of COVID-19 patients presenting AKI, there were no differences in comorbidities between the two gender groups. Nevertheless, compared with females, twice as many male patients needed IV and three times as many male patients needed at least one session of acute dialysis, even if NIPPV and oxygen delivery were used similarly by both genders (Table 1). Males also had SARS and cardiomyopathy more commonly, and their hospital length of stay (median 10 days) was more prolonged, compared with that of females. In addition to antibiotics, male patients also used more antimalarials, steroids, antivirals, other anti-SIRS, and tocilizumab, compared with females (Table 1).

**Figure 1:**
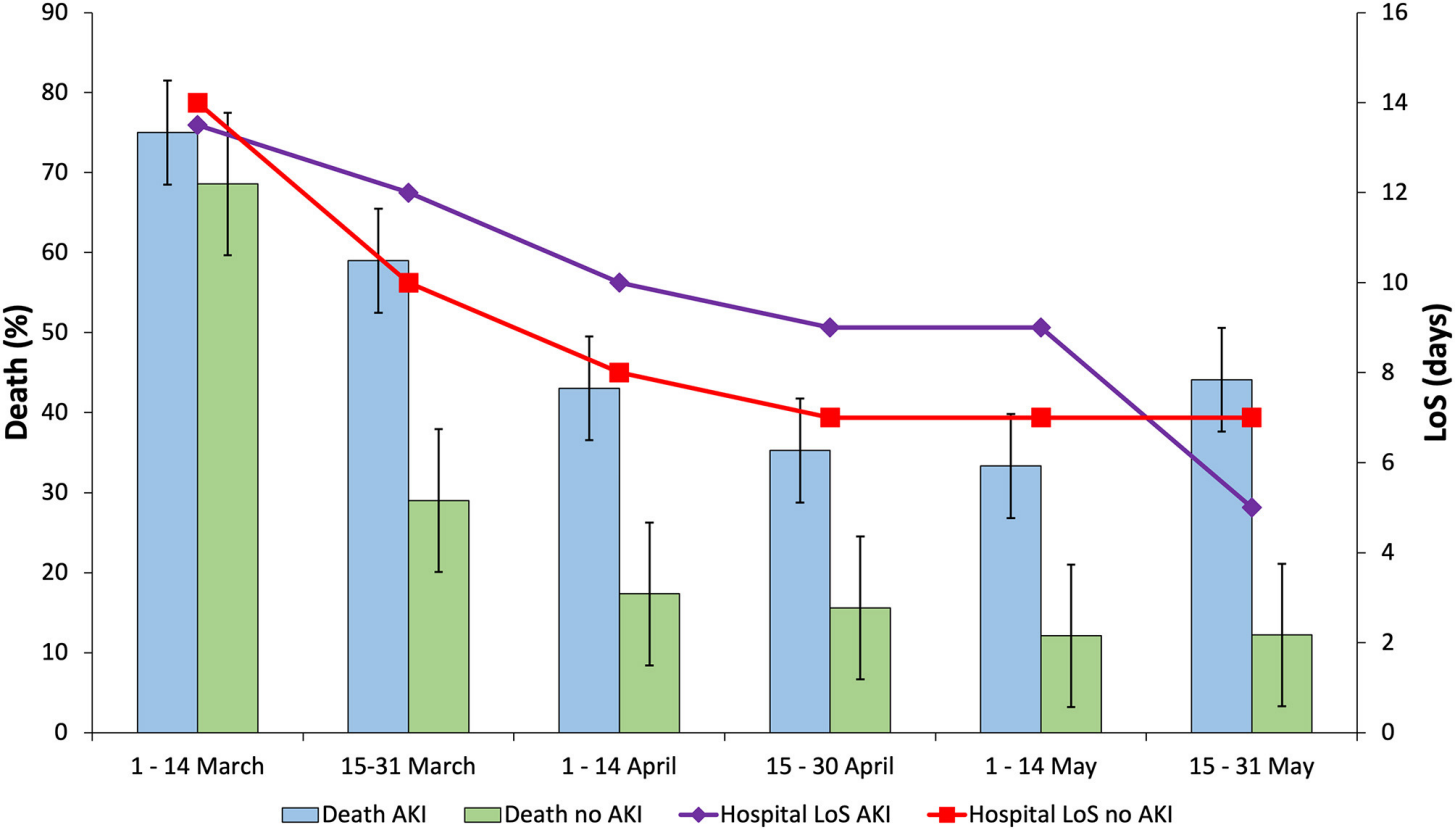
Death and hospital length of stay (LoS) corresponding to the in-hospital COVID-19 patients with or without acute kidney injury (AKI) in Castile and Leon (Spain) (March 1st–May 31th, 2020).

In addition, within the first 15-day period the mortality rate was very high (75%), while decreasing to 44.12% in the last 15-day period. The same trend applies to the length of hospital stay (Figure 1 and Supplementary Table 4). The peak of AKI incidence was reached between April 15th and May 14th (about 14%). COVID-19 patients with AKI needed to stay in the ICU more frequently in the second half of March 2020 (27.2%) (Supplementary Table 4).

### Pharmacological Treatment

**Figure 2:**
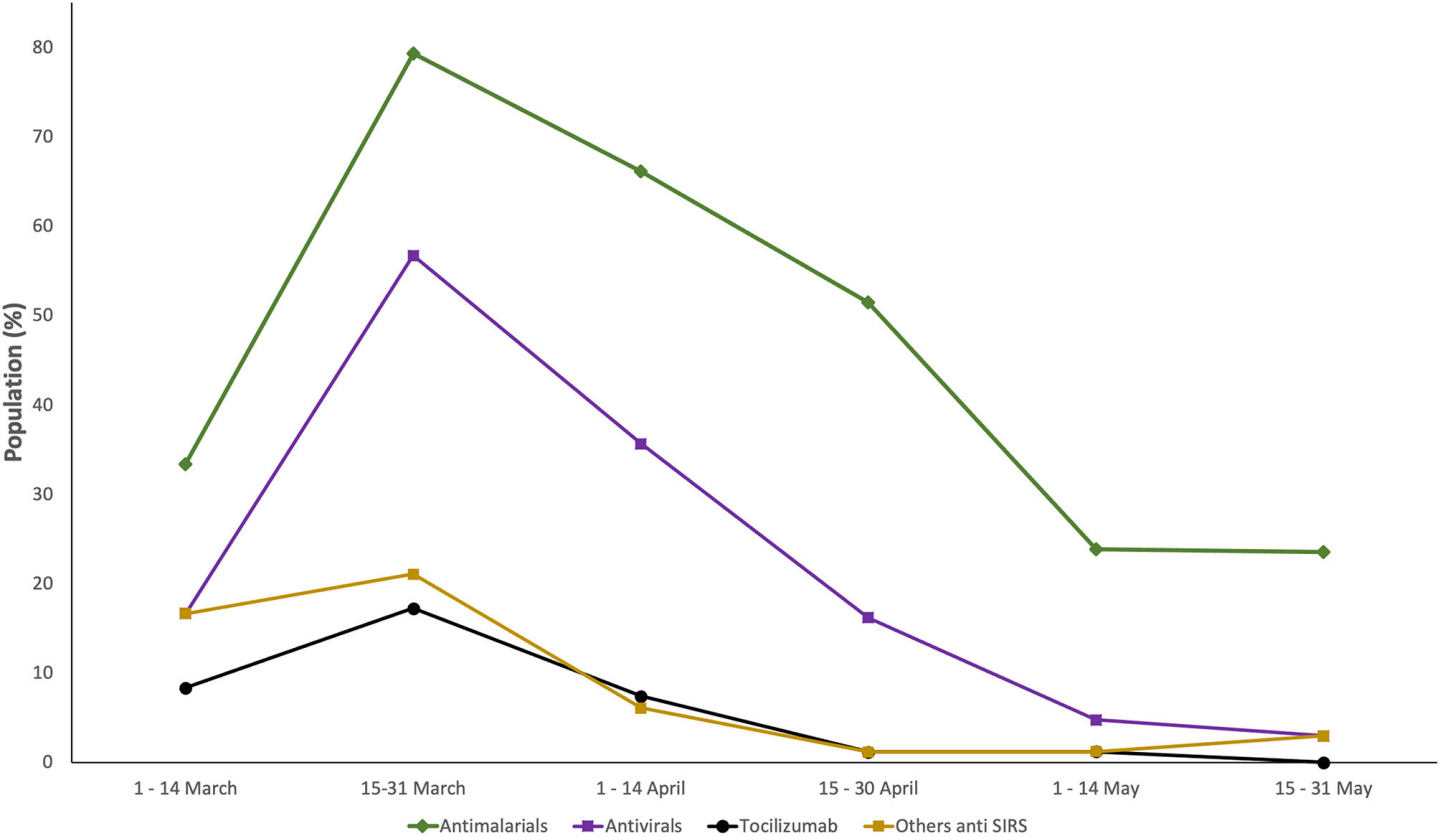
Trends in the use of the medications used by in-hospital COVID-19 patients with acute kidney injury (AKI) in Castile and Leon (Spain) (March 1st–May 31th, 2020).

While the use of antibiotics and steroids, to some extent, remains stable throughout the time, the use of rest of the medications decreased during the study period (Figure 2 and Supplementary Table 4).

Table 2 shows the medication received by COVID-19 patients with AKI who died or survived: significantly lower use of antibiotics and greater use of beta interferon (another anti-SIRS drug category) were noted in those who died compared with those patients who did not die.

**Table 2:** Medications used by in-hospital COVID-19 patients with acute kidney injury (AKI) in Castile and Leon (Spain) (March 1st–May 31th 2020).

**Medications**	**Total**	**No. of deaths**	**No. of deaths**	***p***
	***N* = 794**	***N* = 428**	***N* = 366**	
**Antibiotics**	**90.43 (88.38–92.47)**	**92.76 (90.3–95.21)**	**87.7 (84.34–91.07)**	**0.016**
Ceftriaxone	70.28 (67.1–73.46)	71.5 (67.22–75.77)	68.85 (64.11–73.6)	0.417
Azithromycin	60.71 (57.31–64.1)	62.62 (58.03–67.2)	58.47 (53.42–63.52)	0.233
Levofloxacin	19.14 (16.41–21.88)	19.86 (16.08–23.64)	18.31 (14.34–22.27)	0.579
Teicoplanin	2.27 (1.23–3.3)	2.8 (1.24–4.37)	1.64 (0.34–2.94)	0.272
Cefditoren	1.89 (0.94–2.84)	3.04 (1.41–4.66)	0.55 (0.01–1.08)	0.010
Clarithromycin	0.76 (0.15–1.36)	1.17 (0.15–2.19)	0.27 (0.02–0.53)	0.147
Moxifloxacin	0.25 (0.1–0.4)	0.23 (0.02–0.45)	0.27 (0.02–0.53)	0.912
Cefotaxime	0.25 (0.1–0.4)	0.23 (0.02–0.45)	0.27 (0.02–0.53)	0.912
Ceftaroline	0.13 (0.02–0.23)	0.23 (0.02–0.45)	-	0.355
**Antimalarials**	**60.45 (57.05–63.85)**	**59.58 (54.93–64.23)**	**61.48 (56.49–66.46)**	**0.586**
Hydroxycloroquine	55.54 (52.09–59)	55.37 (50.66–60.08)	55.74 (50.65–60.83)	0.918
Cloroquine	6.55 (4.83–8.27)	5.84 (3.62–8.06)	7.38 (4.7–10.06)	0.383
**Steroids**	**48.61 (45.14–52.09)**	**47.2 (42.47–51.93)**	**50.27 (45.15–55.4)**	**0.387**
Methylprednisolone	45.84 (42.38–49.31)	43.69 (38.99–48.39)	48.36 (43.24–53.48)	0.188
Prednisone	10.33 (8.21–12.44)	13.08 (9.89–16.28)	7.1 (4.47–9.74)	0.006
**Antivirals**	**33.38 (30.1–36.66)**	**31.31 (26.91–35.7)**	**35.79 (30.88–40.7)**	**0.182**
Lopinavir-Ritonavir	33.25 (29.97–36.53)	31.31 (26.91–35.7)	35.52 (30.62–40.42)	0.209
Remdesevir	0.13 (0.02–0.23)	–	0.27 (0.02–0.53)	0.279
**Other anti-SIRS**	**9.45 (7.41–11.48)**	**6.54 (4.2–8.88)**	**12.84 (9.41–16.27)**	**0.010**
Interfereon Beta	8.06 (6.17–9.95)	5.37 (3.24–7.51)	11.2 (7.97–14.43)	0.003
Anakinra	1.26 (0.48–2.04)	0.93 (0.02–1.85)	1.64 (0.34–2.94)	0.375
Ruxolitinib	0.38 (0.05–0.7)	0.23 (0.02–0.45)	0.55 (0.01–1.08)	0.474
Baricitinib	0.25 (0.05–0.44)	0.46 (0.12–0.81)	–	0.190
**Tocilizumab**	**8.31 (6.39–10.23)**	**7.24 (4.79–9.7)**	**9.56 (6.55–12.58)**	**0.238**

*CI, confidence interval; SIRS, systemic inflammatory response syndrome*.

*Values in bold correspond to medicines groups*.

### Survival and Risk Factors for Clinical Outcomes and Medication Prescribed

No impact on survival in patients with or without AKI was observed (median survival: 12 vs. 12 days; *p* = 0.55) (Supplementary Figure 1). However, multiple logistic regression analysis for all in-hospital COVID-19 patients shows the impact of AKI on death (OR: 2.6; 95% CI: 1.73–2.45), as well as comorbidities, such as cardiovascular disease (1.56, 1.37–1.76), diabetes mellitus (1.18, 1.01–1.36), and particularly having an age of 65 years or more (7.34, 5.93–9.08). Furthermore, among the COVID-19 patients with AKI, death was more likely to occur in those requiring ventilation, without distinguishing between invasive and non-invasive modalities (OR: 5.89; 95% CI: 3.13–11.06), in those treated with steroids (1.73, 1.24–2.41) or anti-SIRS drugs (2.38, 1.27–4.44), and in those who developed SARS (2.75, 1.83–4.14) or SIRS (2.52, 1.46–4.34).

## Discussion

This study shows that AKI was present in 1 out of 10 in-hospital COVID-19 patients (March 1st to May 31th, 2020) and that 9 out of 10 cases occurred in people aged more than 65 years, with a mortality around 50%. Ventilation, the use of some medications (steroids, anti-SIRS), and SARS and SIRS incidence may be present in COVID-19 patients with AKI who have an increased risk of death. In addition, AKI had no impact on median survival (in days) compared with in-hospital COVID-19 patients who did not develop AKI. However, mortality among patients with AKI was twice that observed in patients without AKI (46.1 vs. 21.79%) (16), and it is higher than in other studies (17).

Surprisingly, obesity had no influence on death of the patients both with AKI and without AKI, contrary to other studies (18, 19). Probably, lower rates of obesity (in patients with or without AKI, 21.16 and 18.58%, respectively) compared with other cohorts, may be an explanation (20).

Meta-analyses assessing COVID-19 patients from all over the world with AKI confirm that the incidence of AKI was greater in our region compared with Asia (4.3%) but lower than in North America (22.6%) and similar to that of other European countries (11.6%) (3, 8, 21, 22). Surprisingly, the mortality rate in Castile and Leon, Spain, was higher than in all of them. It is difficult to establish which factors are associated with this high mortality rate, which probably is related to the aging of the population in Castile and Leon, but also to other factors, such as the “collapse” of the Spanish public health system, the limited expertise of physicians in treating COVID-19 patients at such a moment, professional extenuation, etc. In this sense, there were differences with respect to other cohorts having an elevated percentage of patients aged 65 years or older, as in our region, which obliging to define the prognosis of the COVID-19 patient with AKI (21): AKI itself seems to have an impact on death, as demonstrated by our analysis and according to findings in other studies (8, 22); however, the impact from other factors should be characterized and considered for different world regions. However, it seems clear that mortality is higher in patients with chronic kidney disease who develop AKI (23).

Our study shows that ventilation, the use of some medicines (steroids, anti-SIRS), the occurrence of SARS or SIRS may depict a patient with a poor prognosis: Physicians should understand that such an individual has an elevated risk of dying, and they should consider these factors in order to assist COVID-19 patients with AKI now and in the future. Importantly, previous clinical conditions, such as age and gender, as well as the need for acute dialysis, seem to have no influence on the change in patients, but the use of some medications (steroids, anti-SIRS) and the severity of COVID-19 (SARS occurrence, signs of SIRS), should lead to more intensive interventions.

This study provides real-world evidence of risk factors associated with the deaths of COVID-19 patients with AKI in the largest region of Spain. As in other healthcare settings (24), assessing data that come from the actual clinical practice has contributed to the characterization of a population suffering bad outcomes. Nevertheless, questions about the quality of the evidence may arise. The findings presented here may be considered to belong to “emerging sources” from outside classic research environments (25).

This real-world data study presents a comprehensive analysis from the COVID-19 pandemic in Castile and Leon, the largest region of Spain, with a population of 2,323,770 inhabitants and a network of public hospitals with a total capacity of 7,141 beds (14 hospitals). Our findings thus cover all in-hospital COVID-19 patients (*n* = 7,307), of whom those presenting with AKI are presented here (*n* = 794).

This study has limitations that should be mentioned. First, although all extracted COVID-19 cases were recorded in the health administration registries accessed as COVID-19 patients, in one third of the cases, COVID-19 diagnosis was clinical or radiological, without microbiological confirmation, as tests were not available for all and because clinical judgment was the only tool at that time. Therefore, risk of selection bias should invite prudence in interpreting the results of this article. Second, due to the collapse of the health system, errors in the clinical data register during the first COVID-19 wave have been observed, which may explain study attrition. Furthermore, selection bias is suspected in the figures of CKD prevalence as not all CKD patients in KDIGO GFR categories 3–5 return to general practitioners after the first consultation in nephrology departments, and not all patients in those CKD categories are sent by general practitioners to the nephrologist. The Angiotensin-Converting Enzyme Inhibitors (ACE)/Angiotensin II Receptor Antagonists (ARB) ratio could be related to the COVID patients' survival; unfortunately, that information was not available, which may be a limitation. Over 8 months have elapsed since the collection of the data, which may be a limitation; however, AKI is still one of the COVID-19 outcomes with the worst prognosis. Finally, other medications not included in Spanish guidelines (14, 15) were not taken into account in this study.

In conclusion, AKI was observed in one 1 out of 10 COVID-19 patients, and almost half of them died before discharge, which demonstrates a mortality rate higher than that observed in other regions including Spain (3, 4, 26–29).

With respect to the pharmacological treatment of these patients, with the exception of antibiotics and steroids, the use of the medications analyzed decreased throughout the study period, either due to their availability or that of other medications not used in our studio. Our study has characterized the subjects hospitalized with COVID-19 and highlighted the use of medicines to treat systemic inflammation, and this situation has not changed up to now.

Lastly, AKI is a serious complication of COVID-19, and it must be taken into account by physicians in order to pay better attention to patients' treatment and to the occurrence of manifestations such as SARS or SIRS associated with a poor prognosis.

## Data Availability Statement

The original contributions presented in the study are included in the article/Supplementary Material, further inquiries can be directed to the corresponding author.

## Ethics Statement

The studies involving human participants were reviewed and approved by East Valladolid Health Area Ethics Committee (reference: PI 20–1863).

## Author Contributions

EG-A, ET, and FÁ: conceptualization. EG-A, FH-G, DM-G, and FÁ: methodology, validation, and investigation. EG-A and ET: software. EG-A, FH-G, ET, and FÁ: formal analysis. FÁ: resources, supervision, project administration, and funding acquisition. EG-A: data curation and visualization. EG-A, FH-G, and FÁ: writing—original draft preparation. EG-A, FH-G, ET, DM-G, and FÁ: writing—review and editing. All authors have read and agreed to the published version of the manuscript.

## Conflict of Interest

The authors declare that the research was conducted in the absence of any commercial or financial relationships that could be construed as a potential conflict of interest.
