# Supplementary material for: Clinical Profile, Pharmacological Treatment, and Predictors of Death Among Hospitalized COVID-19 Patients With Acute Kidney Injury: A Population-Based Registry Analysis
Source: Front Med (Lausanne). 2021 Jun 15;8:657977. doi: 10.3389/fmed.2021.657977 (PMC8240871; doi:10.3389/fmed.2021.657977)
Supplement: Supplementary file 1 [file Data_Sheet_1.docx]

Supplementary Material

# Supplementary Figures and Tables

## Supplementary Figures

**
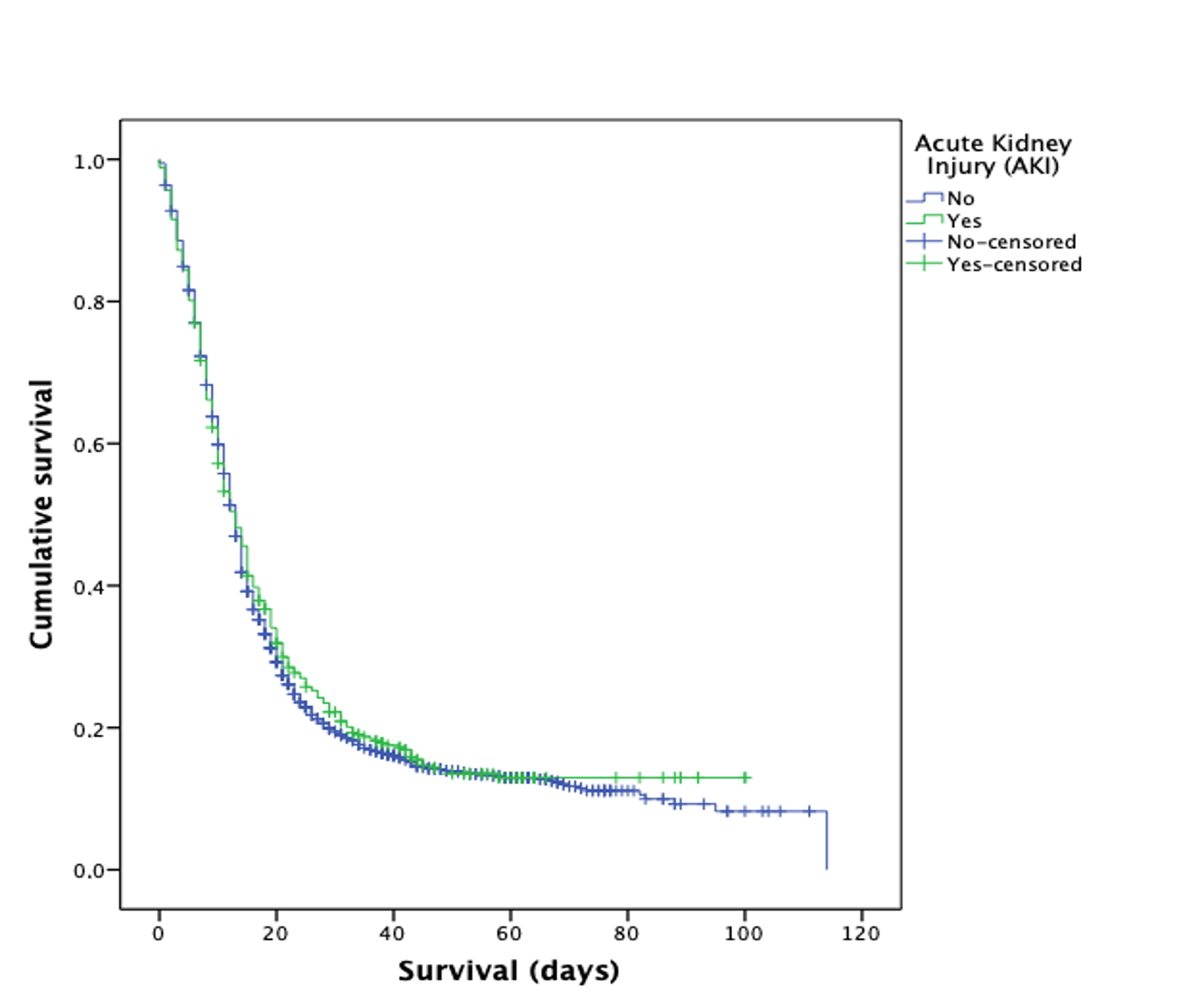
**

**Supplementary Figure 1.** Kaplan-Meier survival curve for in-hospital COVID-19 patients with and without acute kidney injury (AKI) in Castile and Leon (Spain) (March 1^st^ - May 31^th^ 2020).

## Supplementary Tables

**Supplementary Table 1.** Clinical criteria definition of AKI, SIRS and SARS.

| AKI | - Increase in SCr by ≥0.3 mg/dl (≥26.5 mol/l) within 48 hours; or - Increase in SCr to ≥1.5 times baseline, which is known or presumed to have occurred within the prior 7 days; or - Urine volume <0.5 ml/kg/h for 6 hours. |
| --- | --- |
| SIRS | - Temperature: <36°C or >38°C - Heart rate: >90/min - Respiratory rate: >20/min or PaCO2 <32 mmHg (4.3 kPa) - WBC: <4x109/L (<4000/mm³), >12x109/L (>12,000/mm³), or ≥10% bands |
| SARS | - Temperature: >38 °C - Early illness: equal to or more than 2 of the following: chills, rigors, myalgia, diarrhea, sore throat (self-reported or observed) - Mild-to-Moderate illness: indications of lower respiratory tract infection (cough, dyspnea) - Severe Illness: atypical pneumonia, presence of ARDS, autopsy findings in late patients. |

Abbreviations: AKI, acute kidney injury, SCr, serum creatinine, SIRS, systemic inflammatory response syndrome, WBC, white blood cells, SARS, Severe acute respiratory syndrome, ARDS, acute respiratory distress syndrome

**Supplementary Table 2.** COVID-19 pharmacological treatment recommendations according to the Spanish guidelines [14,15].

|  | **Stage I (Early Infection)** | **Stage II (Pulmonary Phase)** | | **Stage III (Hyperinflammation Phase)** |
| --- | --- | --- | --- | --- |
|  |  | **IIa** | **IIb** |  |
| **Clinical Symptoms** | Mild constitutional symptoms  Fever > 99.6 F  Dry Cough, diarrhea, headache | Shortness of breath  Hypoxia (PaO_2_/FiO_2_≤300mmHg) | | ARDS  SIRS/Shock  Cardiac Failure |
| **Clinical Signs** | Lymphopenia, increased prothrombin time, increased D-Dimer and LDH (mild) | Abnormal chest imaging  Transaminitis  Low-normal procalcitonin | | Elevated inflammatory markers  (CRP, LDH, IL-6, D-Dimer, ferritin)  Troponin, NT-proBNP elevation |
| **Potential Therapies** | Lopinavir/Ritonavir 250/50 mg PO every 12 hours only if the clinical course is less than 12 days | Lopinavir/ritonavir 250/50 mg PO every 12 hours only if the clinical course is less than 12 days.  +  Immunomodulatory agent if there is a poor clinical or laboratory course in 24-48 hours. | Lopinavir/ritonavir 250/50 mg PO every 12 hours only if the clinical course is less than 12 days.  +  Acetylcysteine ​​and an immunomodulatory agent. | - Remdesivir if intubation (only in certain hospitals) - Stop Lopinavir/Ritonavir when starting Remdesivir. - Steroids: 3 doses of 250 mg methylprednisolone IV or equivalent on 3 consecutive days. If the patient does not improve, a change to prednisone can be evaluated. |
|  | Consider treatment with antimalarial medicines (preferably hydroxychloroquine)   - Day 1: Hydroxychloroquine 400 mg/12h PO - Day 2 to 5: Hydroxychloroquine 200 mg/12h PO | | | |
| *Immunomodulatory agents* | Option 1: CRP>100 mg/l and ferritin> 1500 mcg/ml, FiO_2_ needs above 35% and procalcitonin less than 0.5 ng/ml. Elevated IL-1, IL-8 and IL-6 and without bacterial superinfection:   - Tocilizumab 400 mg IV. Assess a second infusion at 12 hours or baricitinib 4 mg orally every 24 hours if tocilizumab cannot be used again. - Anakinra 100 mg IV every 6 hours. Consider adding steroids (methylprednisolone 125 mg IV every 24 hours for 3 consecutive days). | | | |
|  | Option 2: CRP>100 mg/ , ferritin<1500 mcg/ml and procalcitonin less than 0.5 ng/ml. Elevated IL-6 and no bacterial superinfection:   - Tocilizumab 400 mg IV. Assess a second infusion at 12 hours or baricitinib 4 mg PO every 24 hours if tocilizumab cannot be used again. | | | |
|  | Option 3: Patient with CRP<100 mg/l, ferritin>1500 mcg/ml, lymphopenia, and procalcitonin<0.5 ng / ml. Elevated IL-1 or IL-8 and no bacterial superinfection:   - Anakinra 100 mg IV every 6 hours. Consider adding steroids (methylprednisolone 125 mg IV every 24 hours for 3 consecutive days). | | | |
| *Antibiotic therapy* | Ceftriaxone with/without Teicoplanin (if superinfection by Gram-positive germ is suspected)  +  Azithromycin or clarithromycin  Cefditoren or Levofloxacin/Moxifloxacin (if allergic to β-lactams) | | Alternative.  Ceftaroline  +  Azithromycin or clarithromycin | |

Abbreviations: ARDS, acute respiratory distress syndrome, SIRS, systemic inflammatory response syndrome, LDH, lactate dehydrogenase, CRP, c-reactive protein, IL, interleukine, NT-proBNP, N-terminal pro hormone B-type natriuretic peptide, PO, orally, IV, intravenous

**Supplementary Table 3**. List of medicines used in the COVID-19 treatment according to Spanish guidelines [14,15].

| **Medicines Type** | **ATC Code** | **Medicine** | **Medicines Type** | **ATC Code** | **Medicine** |
| --- | --- | --- | --- | --- | --- |
| Antibiotics | J01DD01 | Cefotaxime | Anti SIRS Drugs | L01XE18 | Ruxolitinib |
|  | J01DD04 | Ceftriaxone |  | L03AB05 | Interferon alpha 2b |
|  | J01DD16 | Cefditoren |  | L03AB08 | Interferon beta 1b |
|  | J01DI02 | Ceftaroline |  | L04AA37 | Baricitinib |
|  | J01FA09 | Clarithromycin |  | L04AC03 | Anakinra |
|  | J01FA10 | Azithromycin |  | L04AC07 | Tocilizumab |
|  | J01MA12 | Levofloxacin |  | L04AC11 | Siltuximab |
|  | J01MA14 | Moxifloxacin |  | L04AC14 | Sarilumab |
|  | J01XA02 | Teicoplanine | Antivirals | J05AR10 | Lopinavir and Ritonavir |
| Antimalarials | P01BA01 | Chloroquine |  | J05AX95* | Remdesivir |
|  | P01BA02 | Hidroxychloroquine | Steroids | H02AB04 | Methylprednisolone |
|  |  |  |  | H02AB07 | Prednisone |
| Abbreviations: SIRS, systemic inflammatory response syndrome | | | |  |  |

**Supplementary Table 4**. Treatment and clinical outcomes evolution of in-hospital COVID-19 patients with acute kidney injury in Castile and Leon (Spain) (March 1^st^ - May 31^th^ 2020).

| **Medicines (% 95 CI)** | **1-14 March** | **15-31 March** | **1-14 April** | **15-30 April** | **1-14 May** | **15-31 May** |
| --- | --- | --- | --- | --- | --- | --- |
|  | N=12 | N=261 | N=230 | N=173 | N=84 | N=34 |
| **Antibiotics** | **66.67 (39.99-93.34)** | **90.8 (87.3-94.31)** | **90.87 (87.15-94.59)** | **90.17 (85.74-94.61)** | **90.48 (84.2-96.75)** | **94.12 (86.21-102.03)** |
| Ceftriaxone | 58.33 (30.44-86.23) | 68.58 (62.95-74.21) | 70.87 (65-76.74) | 68.79 (61.88-75.69) | 76.19 (67.08-85.3) | 76.47 (62.21-90.73) |
| Azithromycin | 16.67 (0.51-33.75) | 65.52 (59.75-71.28) | 63.48 (57.26-69.7) | 59.54 (52.22-66.85) | 51.19 (40.5-61.88) | 50 (33.19-66.81) |
| Levofloxacin | 25 (0.5-49.5) | 22.99 (17.88-28.09) | 15.22 (10.58-19.86) | 18.5 (12.71-24.28) | 21.43 (12.65-30.2) | 11.76 (0.93-22.59) |
| Clarithromycin | - | - | - | 1.73 (0.21-3.26) | - | 8.82 (0.71-16.96) |
| Teicoplanin | - | 3.07 (0.97-5.16) | 3.04 (0.82-5.26) | 1.16 (0.12-2.21) | 1.19 (0.13-2.51) | - |
| Cefditoren | - | 1.92 (0.25-3.58) | 2.61 (0.55-4.67) | 2.31 (0.07-4.55) | - | - |
| Cefotaxime | - | - | - | 0.58 (0.05-1.23) | 1.19 (0.13-2.51) | - |
| Moxifloxacin | - | 0.38 (0.02-0.87) | 0.43 (0.08-1.13) | - | - | - |
| Ceftaroline | - | - | - | 0.58 (0.05-1.23) | - | - |
| **Antimalarials** | **33.33 (6.66-60.01)** | **79.31 (74.4-84.22)** | **0.43 (0.08-1.13)** | **51.45 (44-58.89)** | **23.81 (14.7-32.92)** | **23.53 (9.27-37.79)** |
| Hydroxycloroquine | 25 (0.5-49.5) | 70.88 (65.37-76.39) | 60.43 (54.12-66.75) | 50.29 (42.84-57.74) | 22.62 (13.67-31.57) | 23.53 (9.27-37.79) |
| Cloroquine | 8.33 (0.3-15.97) | 13.41 (9.28-17.54) | 5.65 (2.67-8.64) | 1.16 (0.12-2.21) | 1.19 (0.13-2.51) | - |
| **Steroids** | **66.67 (39.99-93.34)** | **48.66 (42.6-54.72)** | **48.7 (42.24-55.16)** | **50.29 (42.84-57.74)** | **40.48 (29.98-50.97)** | **52.94 (36.16-69.72)** |
| Methylprednisolone | 66.67 (39.99-93.34) | 46.36 (40.31-52.41) | 44.78 (38.36-51.21) | 47.98 (40.53-55.42) | 36.9 (26.59-47.22) | 52.94 (36.16-69.72) |
| Prednisone | 16.67 (0.51-33.75) | 10.73 (6.97-14.48) | 11.74 (7.58-15.9) | 7.51 (3.59-11.44) | 10.71 (4.1-17.33) | 8.82 (0.71-16.96) |
| **Antivirals** | **16.67 (0.51-33.75)** | **56.7 (50.69-62.72)** | **35.65 (29.46-41.84)** | **16.18 (10.7-21.67)** | **4.76 (0.21-9.32)** | **2.94 (0.74-6.62)** |
| Lopinavir-Ritonavir | 16.67 (0.51-33.75) | 56.7 (50.69-62.72) | 35.22 (29.04-41.39) | 16.18 (10.7-21.67) | 4.76 (0.21-9.32) | 2.94 (0.74-6.62) |
| Remdesevir | - | - | 0.43 (0.08-1.13) | - | - | - |
| **Others anti SIRS** | **16.67 (0.51-33.75)** | **21.07 (16.13-26.02)** | **6.09 (3-9.18)** | **1.16 (0.12-2.21)** | **1.19 (0.13-2.51)** | **2.94 (0.74-6.62)** |
| Interferon Beta | 16.67 (0.51-33.75) | 20.31 (15.43-25.19) | 3.91 (1.41-6.42) | - | - | - |
| Anakinra | - | 0.77 (0.13-1.33) | 2.17 (0.29-4.06) | 1.16 (0.12-2.21) | - | 2.94 (0.74-6.62) |
| Baricitinib | - | 0.38 (0.02-0.87) | - | - | 1.19 (0.13-2.51) | - |
| Ruxolitinib | - | 1.15 (0.14-2.30) | - | - | - | - |
| **Tocilizumab** | 8.33 (0.3-15.97) | 17.24 (12.66-21.82) | 7.39 (4.01-10.77) | 1.16 (0.12-2.21) | 1.19 (0.13-2.51) | - |
| Hospital LoS (median + IQR) | 13.50 (4.00-31.50) | 12.00 (5.00-23.00) | 10.00 (5.00-18.00) | 9.00 (6.00-17.00) | 9.00 (6.00-15.00) | 5.00 (3.00-8.00) |
| ICU LoS (median + IQR) | 19.00 (19.00-19.00) | 14.00 (7.00-25.00) | 13.00 (4.00-19.00) | 7.50 (2.00-34.00) | 11.50 (9.50-28.00) | - |
|  | N=1 | N=71 | N=19 | N=4 | N=4 | N=0 |
| Death (% 95 CI) | 75 (50.5-99.5) | 59 (53.04-64.97) | 43.04 (36.64-49.44) | 35.26 (28.14-42.38) | 33.33 (23.25-43.41) | 44.12 (27.43-60.81) |
| SARS (% 95 CI) | 8.33 (0.3-15.97) | 37.16 (31.3-43.03) | 23.48 (18-28.96) | 13.87 (8.72-19.02) | 13.1 (5.88-20.31) | 14.71 (2.8-26.61) |
| Bacterial superinfection (% 95 CI) | 16.67 (0.51-33.75) | 9.2 (5.69-12.7) | 11.74 (7.58-15.9) | 20.23 (14.24-26.22) | 20.24 (11.65-28.83) | 14.71 (2.8-26.61) |
| SIRS (% 95 CI) | 16.67 (0.51-33.75) | 8.81 (5.37-12.25) | 10.43 (6.48-14.39) | 13.29 (8.24-18.35) | 9.52 (3.25-15.8) | 14.71 (2.8-26.61) |
| Fungal superinfection (% 95 CI) | 8.33 (0.3-15.97) | 7.28 (4.13-10.43) | 6.52 (3.33-9.71) | 4.62 (1.49-7.75) | 3.57 (0.4-7.46) | 2.94 (0.74-6.62) |
| Carodiomyopathy (% 95 CI) | - | 1.92 (0.25-3.58) | 3.91 (1.41-6.42) | 3.47 (0.74-6.19) | 3.57 (0.4-7.46) | - |
| DIC (% 95 CI) | - | 1.53 (0.04-3.02) | 0.43 (0.08-1.13) | 0.58 (0.05-1.23) | 1.19 (0.13-2.51) | 2.94 (0.74-6.62) |

Abbreviations: 95 CI, confidence interval, SIRS, systemic inflammatory response syndrome, ICU, intensive care unit, LoS, length of stay, IQR, interquartile range, SARS, severe acute respiratory syndrome, DIC, disseminated intravascular coagulation.
